# Extranodal NK/T‐Cell Lymphoma in the Nasal Region Illustrating the Differential Diagnosis of Lesions of the Face

**DOI:** 10.1002/ccr3.72740

**Published:** 2026-05-18

**Authors:** Leonor Dias, Henrique Messias, Carlos Zagalo, Carla Alves, Gonçalo Esteves, Pedro Gomes

**Affiliations:** ^1^ Faculdade de Medicina da Universidade de Lisboa Lisbon Portugal; ^2^ Serviço de Cirurgia de Cabeça e Pescoço, Instituto Português de Oncologia de Lisboa Francisco Gentil, Centro Clínico Académico de Lisboa (CCAL) Lisbon Portugal; ^3^ Division of Health Sciences University of Edinburgh Edinburgh UK; ^4^ Serviço de Anatomia Patológica, Instituto Português de Oncologia de Lisboa Francisco Gentil, Centro Clínico Académico de Lisboa (CCAL) Lisbon Portugal

**Keywords:** Epstein–Barr virus, extranodal NK/T‐cell lymphoma, head and neck neoplasms, lymphoma, oncogenic viruses

## Abstract

Extranodal NK/T‐cell lymphoma is an aggressive malignancy, rare in Western populations, that can mimic benign diseases. Given the impact that prompt diagnosis has on the treatment and prognosis, it should be included in the differential diagnosis of lesions presenting in the upper aerodigestive tract, particularly the nasal region.

## Graphical abstract

Histopathological analysis of an infiltrative nasal lesion, diagnostic for Extranodal NK/T cell lymphoma (ENKTL), with its characteristic angiotropism, angiodestruction, and immunohistochemical staining, including positivity for EBER.

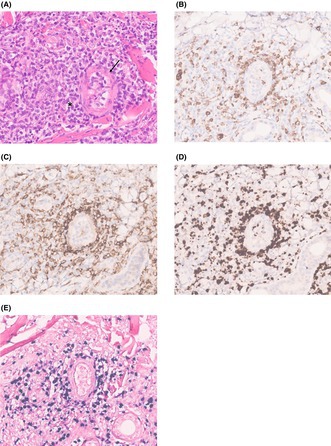

## 1 Introduction

Extranodal natural killer/T‐cell lymphoma (ENKTL) is an aggressive lymphoproliferative malignancy associated with Epstein–Barr virus (EBV) infection and extranodal involvement, classically affecting the upper aerodigestive tract. It predominantly affects adult males between 46 and 60 years of age. It is common in Asia and South America, but extremely rare in Europe and North America. The reason for this geographic prevalence diversity is incompletely understood but is probably due to circulating EBV strains and population genetic susceptibility [1, 2, 3].

The clinical presentation often mimics infectious and inflammatory processes, and histological evaluation is further complicated by the extensive necrosis typically found in the lesions. Consequently, diagnosis of this disease is frequently challenging, particularly in Western populations where its prevalence is low and in pediatric patients [1, 4].

Over the past two decades, significant therapeutic advances have been achieved, including improvements in radiotherapy techniques, the introduction of L‐asparaginase (L‐Asp)–based chemotherapy regimens, and the use of immunotherapy with immune checkpoint inhibitors targeting PD‐1/PD‐L1 [4, 5, 6].

The authors report a case of ENKTL of the nasal region, illustrating the diagnostic challenges associated with this entity. Thorough histopathological evaluation proved essential for the identification of this lymphoma, which is exceedingly rare in Portugal.

## 2 Case History/Examination

A 60‐year‐old caucasian male patient was referred to the Head and Neck Surgery Department with a three‐month history of an ulcerative cutaneous lesion on the right hemiface, extending from the infraorbital region to the mandibular line, with an infiltrative component involving the ipsilateral nasal cavity. The lesion was associated with inflammatory signs and had shown progressive enlargement over this period. The patient also reported a 13% loss of body weight during the same timeframe. There was no relevant past medical history or family history. Remaining head and neck and general physical examination was unremarkable.

## 3 Differential Diagnosis, Investigations and Treatment

**FIGURE 1:**
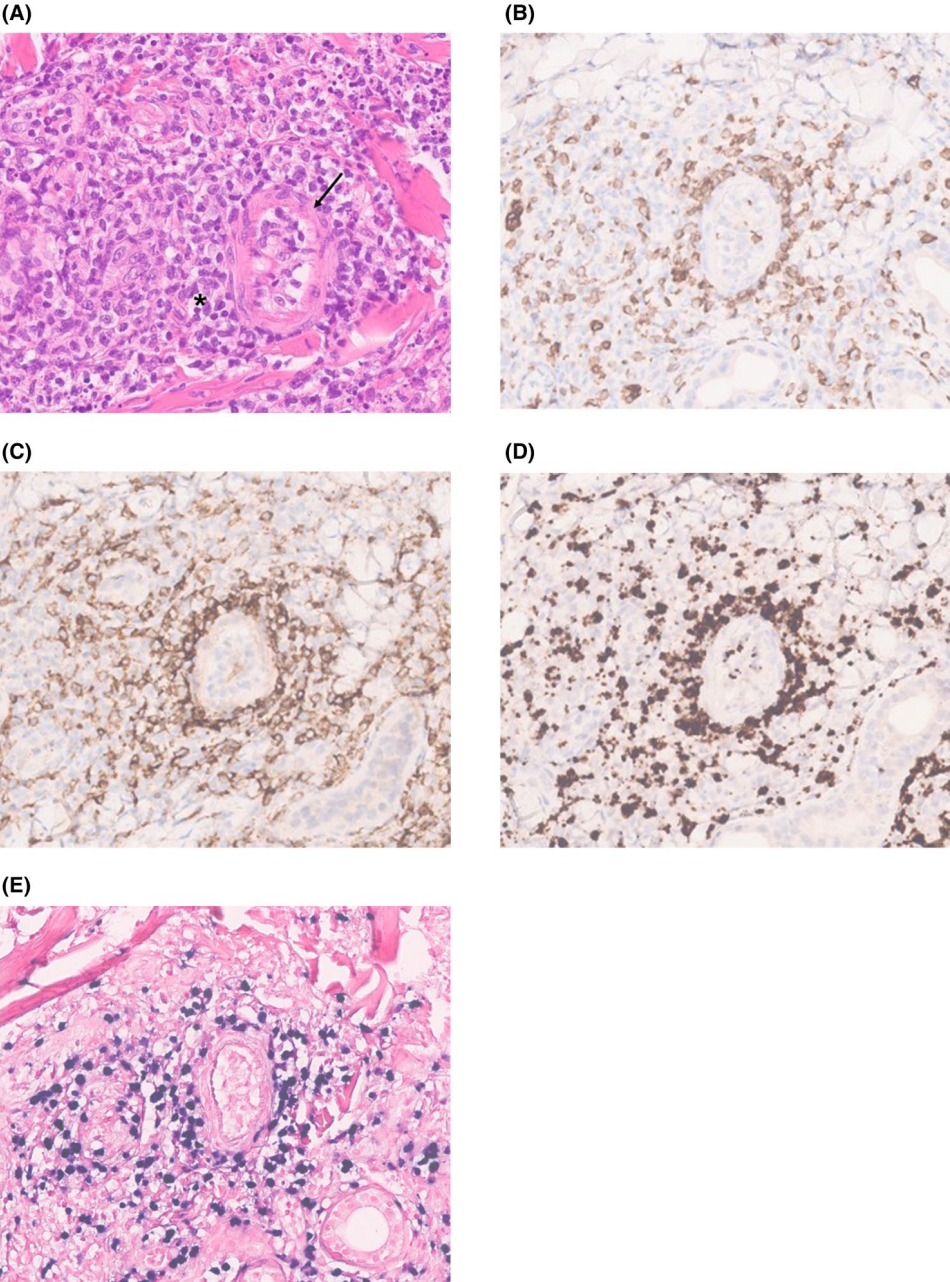
Extranodal natural killer/T‐cell lymphoma (ENKTL). Histopathological evaluation of the biopsy from the soft tissues of the right hemiface. Diffuse infiltration by small lymphocytes, with irregular nuclei (H&E: A, asterisk). Angiotropism and angio‐destruction of small vessels (A, arrow). Figures (B–D) show immunohistochemical staining characteristic of the NK‐cell lineage: Cytoplasmic CD3 positivity (B); CD56 positivity (C); granzyme B positivity (D). EBER positivity (E).

An incisional biopsy of the lesion was performed, and the final diagnosis was of an ENKTL. The histopathological report stated that the neoplastic cells expressed CD3 (cytoplasmic), CD2, CD56, CD30 (20%, weak), TIA1, and granzyme B, and were negative for CD20, CD5, CD7, and CD8. In addition, Epstein–Barr virus–encoded RNA (EBER), detected by in situ hybridization, was present in all tumor cells (Figure 1).

**FIGURE 2:**
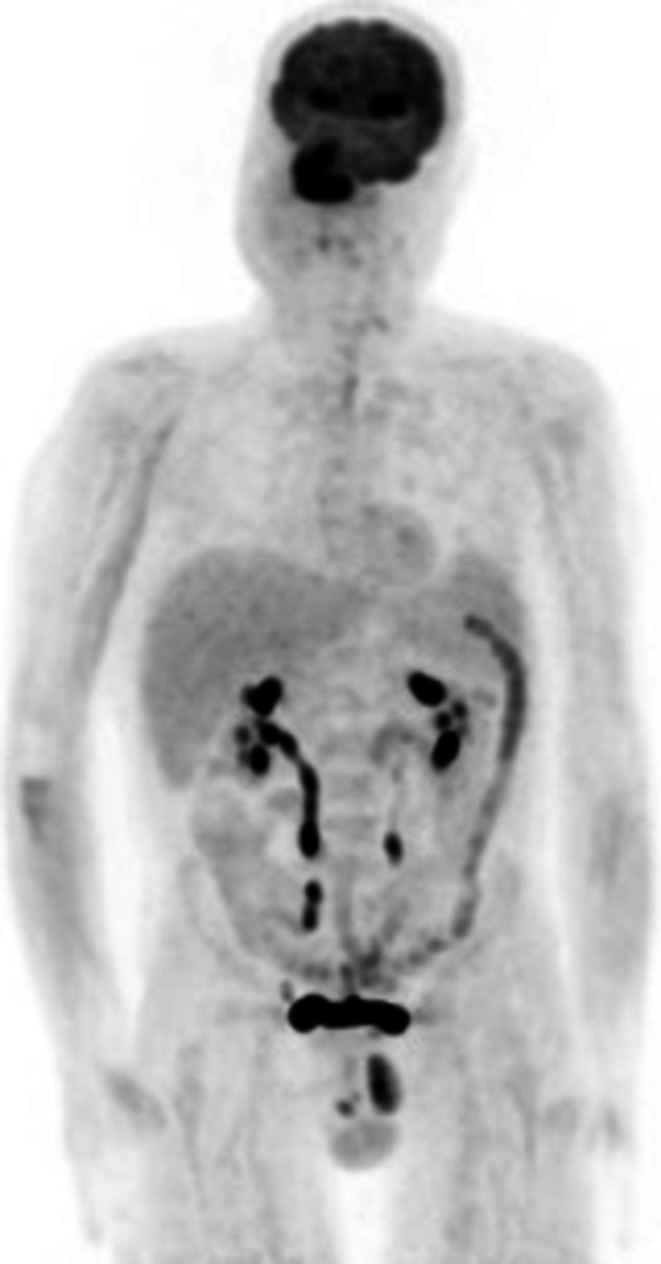
ENKTL of the nasal region. Whole‐body FDG‐PET study.

For this reason, the patient was referred to the Hematology Department. A whole‐body Positron Emission Tomography with Fluorodeoxyglucose (PET‐FDG) scan was performed, which revealed abnormal radiotracer uptake consistent with lymphoma at the nasal region (right ala and septum, and surrounding subcutaneous tissue and skin), as well as the right paranasal and infraorbital regions (Figure 2).

A bone marrow biopsy and aspirate were also performed, revealing a slightly hypercellular, reactive bone marrow with preserved hematopoietic maturation and no evidence of neoplastic infiltration.

**FIGURE 3:**
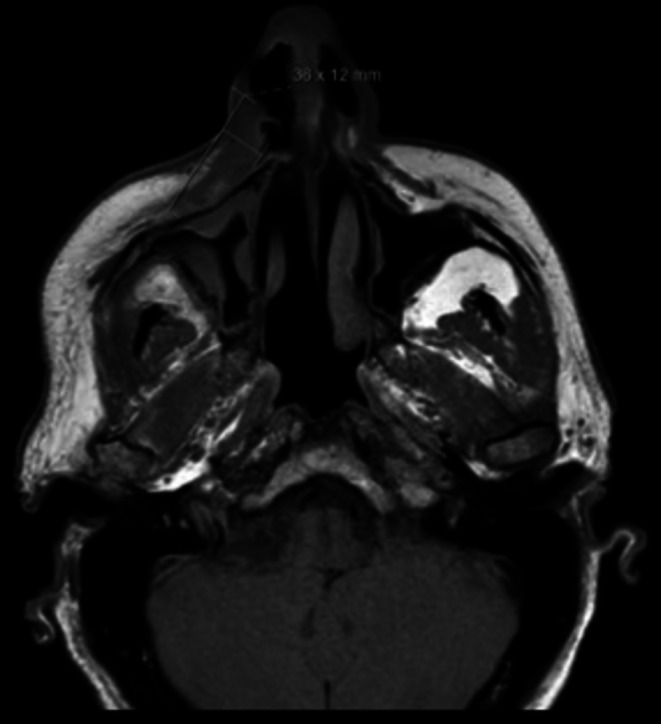
ENKTL of the nasal region. Axial T1‐weighted magnetic resonance imaging (MRI).

Finally, a maxillofacial MRI was obtained, demonstrating thickening of the skin and subcutaneous tissue in the right cheek and nasolabial fold, measuring approximately 36 × 12 mm in maximal axial dimensions (Figure 3). The lesion showed no significant contrast enhancement but exhibited an area of restricted diffusion. Although the lesion was near the right infraorbital foramen, no signs of perineural spread were identified.

The case was staged based on the Lugano system as IE disease with risk factors. After discussion at the Multidisciplinary Lymphoma Board, the patient was referred and then completed systemic treatment, consisting of three cycles of P‐GemOx (with PEG‐asparaginase 1500 IU/m^2^), followed by radiotherapy (RT) to a total dose of 50 Grays in 25 fractions, and finally three additional cycles of GemOx. During the last three chemotherapy (ChT) cycles, PEG‐asparaginase was omitted due to the patient's refusal following an episode of anaphylaxis to this drug on the first day of cycle 4.

## 4 Conclusions and Results

Throughout the first three cycles of ChT, there was a progressive reduction of the facial lesion and edema, with resolution of associated pain. At the end of RT, right‐sided nasal obstruction developed, mainly due to collapse of the right alar cartilage, with an associated paramedian oronasal fistula.

After completing the fourth ChT cycle, the patient developed redness of the right eye, eyelid edema, and periorbital pain. Nasal endoscopy revealed marked endonasal mucositis. A contrast‐enhanced CT scan of the paranasal sinuses showed no abscess formation or signs of orbital cellulitis. Due to the suspicion of invasive fungal sinusitis in a high‐risk immunocompromised patient, treatment was initiated with antibiotics (4 days of intravenous ceftriaxone followed by oral cefuroxime 500 mg b.i.d. for a total of 3 weeks) and antifungal therapy (oral isavuconazole 100 mg b.i.d. for a total of 6 weeks). There was clinical improvement but persistence of epiphora and nasal obstruction. Another CT scan of the paranasal sinuses was performed to assess the need for surgical drainage or biopsy to exclude refractory disease; neither was indicated.

After completion of treatment, a whole‐body PET‐FDG scan demonstrated metabolic improvement of the lesion on the right hemiface, but persistence of a small area of diffuse uptake near the anterior nasal spine, not allowing exclusion of residual disease. A maxillofacial CT scan was requested for better characterization and biopsy guidance. An incisional biopsy of this area, performed through the oral cavity, showed no evidence of disease.

**FIGURE 4:**
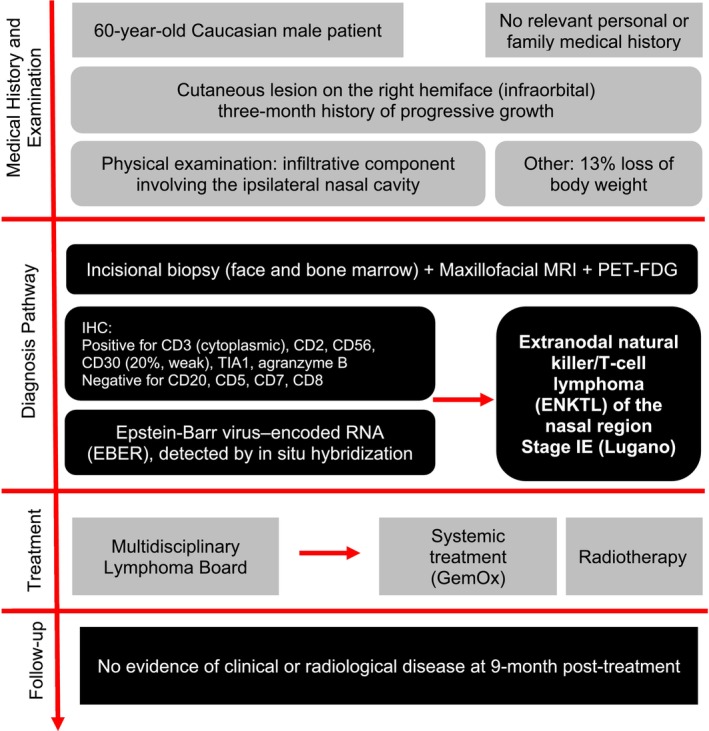
Detailed diagram of the patient's case timeline.

At 9 months of follow‐up there were no clinical or radiologic signs of disease recurrence. (Figure 4).

In conclusion, ENKTL mimics a wide range of clinical entities both clinically and histologically. It should be included in the differential diagnosis of infiltrative and ulcerative lesions of the upper aerodigestive tract, including in populations in Western countries where its prevalence is low. Raising awareness in the medical and scientific community is crucial, as delayed diagnosis and treatment initiation have significant prognostic implications.

## 5 Discussion

The term “Extranodal natural killer/T‐cell lymphoma (ENKTL)” was updated in the most recent (5th edition) of the World Health Organization Classification of Haematolymphoid Tumors, with the qualifier “nasal‐type” dropped from its name, in accordance with the recognized presentation of this disease at various extranodal sites [7]. Clinically, ENKTL can be subdivided into two distinct subtypes based on the sites involved: nasal and non‐nasal [8]. The most common, accounting for approximately 80% of cases, is the nasal subtype, which involves the upper aerodigestive tract and is frequently localized at diagnosis [1, 5]. Also, the primary lesion in nasal‐subtype disease is typically a necrotic ulcer with progressive evolution, initially presenting with symptoms such as nasal obstruction, rhinorrhea, epistaxis, odynophagia, and dysphonia. If not treated promptly, the disease frequently extends to adjacent tissues, causing extensive regional destruction. In this case, the initial manifestation was a progressively enlarging ulcerative lesion already extending to adjacent soft tissues of the face. Additionally, B symptoms are frequently observed; in this patient, there was significant weight loss [1, 9].

In a patient presenting with an ulcerative and infiltrative facial lesion extending into the nasal cavity, the initial differential diagnoses include infectious diseases, granulomatous disorders, and neoplasms (Table 1). Lymphomas are the third most common sinonasal neoplasm after squamous cell carcinoma and adenocarcinoma, representing 12%–15% of head and neck malignancies [1]. Endoscopic evaluation of the upper airway and histologic assessment via lesion biopsy are paramount for a definitive diagnosis.

**TABLE 1:** Differential diagnoses for ENKTL of the nasal region [1].

Differential diagnoses of nasal type extranodal NK/T‐cell lymphoma (ENKTL)	
Infectious diseases	Rhinoscleroma Facial cellulitis Fungal sinusitis Leishmaniasis Tertiary syphilis
Granulomatous diseases	Granulomatosis with polyangiitis (formerly Wegener's granulomatosis)
Neoplasms	Squamous cell carcinoma Adenocarcinoma Lymphomas (Lymphomatoid granulomatosis, nasal‐type ENKTL)

*Note:* Use of inhaled drugs (particularly cocaine).

Excisional or incisional biopsy is preferred over core‐needle biopsy, as it provides sufficient tissue for histologic evaluation [8, 9]. As it was our case, an incisional biopsy of the facial soft tissues was performed, as the lesion had already extended to this site. Necrosis is commonly observed in biopsy specimens and may significantly delay diagnosis by interfering with histopathologic interpretation. Some strategies that help to maximize the likelihood of obtaining viable tissue include margins of uninvolved tissue, collecting multiple biopsies from the nasopharynx, including areas that appear uninvolved, and collecting biopsies from the palatal mucosa when it is involved [1, 10, 11, 12]. Histologically, the lesion consists of a diffuse infiltrate of atypical lymphoid cells (spanning a phenotype from NK to T cells, hence the designation) exhibiting an angiodestructive growth pattern, resulting in ischemia and characteristic necrosis (notably perivascular hyaline and geographic necrosis), which leads to ulceration. This angiocentric and angiodestructive feature was historically so prominent that the entity was once termed angiocentric lymphoma [1, 9].

ENKTL is strongly associated with EBV infection. Following entry into lymphocytes, EBV persists by expressing a range of viral genes in distinct latency programs. One clinically relevant product is EBER, present in all EBV‐infected cells across latency programs. EBER transcripts are the most abundant viral transcripts, with over one million copies per infected cell. For these reasons, in situ hybridization (ISH) for EBER remains the gold standard for detecting EBV‐infected cells [13]. Thus, diagnosis requires confirmation of EBV positivity in neoplastic cells via EBER ISH. Additionally, neoplastic cells should express CD56 (an NK‐cell lineage marker) or cytotoxic molecules (granzyme B, perforin, TIA1) by immunohistochemistry. These were also verified in the present case. Absence of CD20 is also important, as it excludes B‐cell lineage, aiding differentiation from B‐cell lymphomas [1, 9, 14].

Staging is performed according to the Lugano classification, derived from the Ann Arbor system, currently the most widely used for ENKTL [3]. PET‐FDG is essential, as ENKTL is typically FDG‐avid, allowing detection of subclinical metastases [10, 11]. Bone marrow biopsy or aspirate may assess marrow involvement, indicative of disseminated disease. Some authors suggest that in early‐stage disease, bone marrow evaluation may be omitted in favor of PET‐FDG alone [1]. (Table 2) A recent risk stratification model, the Prognostic Index for Natural Killer Lymphoma (PINK), uses four clinical parameters associated with poorer overall survival (OS): age > 60 years, stage III/IV, distant lymph node involvement, and non‐nasal disease. Patients are classified as low‐risk (no risk factors), intermediate‐risk (1 risk factor), and high‐risk (≥ 2 risk factors), with 3‐year OS of 81%, 62%, and 25%, respectively [1, 10]. Based on this model, the presented patient falls into the intermediate‐risk group, due to age.

**TABLE 2:** Recommended approach after ENKTL of the nasal region. Adapted from the NCCN Guidelines for T‐cell Lymphomas (v2.2025) [8].

Recommended approach after the diagnosis of nasal‐type extranodal NK/T‐cell lymphoma (ENKTL)
**Clinical history—**assess for B symptoms and determine performance status
**Physical examination**–with special attention to regions containing lymph nodes (such as Waldeyer's ring), testes, and skin
**Endoscopic evaluation of the upper airway**
**Laboratory tests** Complete blood count with differential; Comprehensive biochemistry panel (urea, creatinine, electrolytes, bicarbonate, aminotransferases, alkaline phosphatase, total bilirubin, albumin, total proteins, fasting glucose); Lactate dehydrogenase (LDH) and uric acid
PET‐FDG associated with CT and/or contrast‐enhanced thoraco‐abdomino‐pelvic CT
**Bone marrow aspiration and biopsy**
**Calculate prognostic index** (PINK or PINK‐E)
**Echocardiogram** or equilibrium radionuclide angiography, if chemotherapy with anthracyclines is planned
**EBV viral load** (by PCR)
**Pre‐treatment radiotherapy evaluation with MRI ± CT for RT planning**
**Pregnancy test** if of childbearing age and chemotherapy or radiotherapy is planned
**In certain circumstances, additional tests may be useful:** Screening for infections (HIV, hepatitis B, hepatitis C, and HTLV‐1/2) Ophthalmologic evaluation Lumbar puncture and cerebrospinal fluid analysis Discussion with the patient regarding fertility preservation

The optimal therapeutic approach for ENKTL remains undefined [1, 2, 5]. As a rare disease, few randomized clinical trials compare treatment regimens; most evidence comes from retrospective studies or small prospective series [1, 10]. The treatment protocol used aligns with the most recent NCCN guidelines for T‐cell lymphomas (May 28, 2025) and ESMO guidelines for peripheral T‐cell and NK‐cell lymphomas (January 23, 2025). For stage I–IIE disease, considered limited, RT is critical for local disease control. The total RT dose is crucial; as recommended by ESMO, the patient received ≥ 50 Gy [1, 5, 10, 11]. Given the patient's performance status (ECOG < 2), chemotherapy (CT) was administered using the P‐GemOx regimen (PEG‐asparaginase, gemcitabine, oxaliplatin), a less intensive regimen compared to others used for ENKTL. ENKTL exhibits metabolic vulnerability to L‐asparaginase, making it a key component of chemotherapy regimens [1, 5, 10, 11].

After treatment, follow‐up includes repeating imaging, nasal endoscopy, and plasma EBV‐DNA PCR. Outcomes are classified as complete response, partial response, no response/stable disease, or progressive disease. In this case, the patient achieved complete response [10, 11].

Awareness of treatment‐related toxicities is essential for short‐ and long‐term patient management. Acute RT toxicities are generally transient, dose‐dependent, and confined to the irradiated region. Late RT toxicities, though rare, include cerebrospinal fluid fistula, nasal obstruction, and chronic sinusitis. Chemotherapy toxicities depend on the regimen but commonly include hematologic, hepatic, and renal effects and increased infection risk [14, 15].

A recent multicenter study of early‐stage ENKTL showed that chemoradiotherapy in intermediate‐risk patients, as in this case, resulted in 5‐year overall survival of 77.7% and 5‐year progression‐free survival of 67.1% [15].

## Author Contributions

**Leonor Dias:** conceptualization, data curation, formal analysis, investigation, methodology, project administration, resources, writing – original draft, writing – review and editing. **Henrique Messias:** conceptualization, data curation, funding acquisition, project administration, validation, visualization, writing – original draft, writing – review and editing. **Carlos Zagalo:** conceptualization, data curation, investigation, project administration, software, supervision, writing – review and editing. **Carla Alves:** data curation, methodology, resources, software. **Gonçalo Esteves:** resources, software, validation, visualization, writing – review and editing. **Pedro Gomes:** project administration, resources, supervision, validation, visualization.

## Funding

The authors declare that they have received funding for publication charges from their health care institution (Instituto Português de Oncologia de Lisboa Francisco Gentil, Lisboa, Portugal).

## Consent

Written informed consent was obtained from the patient.

## Conflicts of Interest

The authors declare no conflicts of interest.

## Data Availability

The authors declare that all data supporting the findings of this study are available within the article and its supporting information files.
